# *Undaria pinnatifida* and Fucoxanthin Ameliorate Lipogenesis and Markers of Both Inflammation and Cardiovascular Dysfunction in an Animal Model of Diet-Induced Obesity

**DOI:** 10.3390/md14080148

**Published:** 2016-08-03

**Authors:** Ameyalli Grasa-López, Ángel Miliar-García, Lucía Quevedo-Corona, Norma Paniagua-Castro, Gerardo Escalona-Cardoso, Elba Reyes-Maldonado, María-Eugenia Jaramillo-Flores

**Affiliations:** 1 Departamento de Ingeniería Bioquímica, Escuela Nacional de Ciencias Biológicas, Instituto Politécnico Nacional, Ciudad de México 07738, Mexico; ameyalli.grasa@gmail.com; 2 Laboratorio de Biología Molecular, Escuela Superior de Medicina, Instituto Politécnico Nacional, Ciudad de México 11340, Mexico; angel.miliar@yahoo.com.mx; 3 Departamento de Fisiologia, Escuela Nacional de Ciencias Biológicas, Instituto Politécnico Nacional, Ciudad de México 07738, Mexico; luciaquevedoc@gmail.com (L.Q.-C.); npaniag@hotmail.com (N.P.-C.); gerescalona@yahoo.com.mx (G.E.-C.); 4 Departamento de Morfología, Escuela Nacional de Ciencias Biológicas, Instituto Politécnico Nacional, Ciudad de México 11340, Mexico; elbareyesm@gmail.com

**Keywords:** fucoxanthin, *Undaria pinnatifida*, obesity, inflammation, energy expenditure, adipose tissue expansion, gene expression

## Abstract

Brown algae and its carotenoids have been shown to have a positive influence on obesity and its comorbidities. This study evaluated the effect of *Undaria pinnatifida* and fucoxanthin on biochemical, physiological and inflammation markers related to obesity and on the expression of genes engaged on white adipose tissue lipid metabolism in a murine model of diet-induced obesity. The treatments improved energy expenditure, β-oxidation and adipogenesis by upregulating PPARα, PGC1α, PPARγ and UCP-1. Adipogenesis was also confirmed by image analysis of the retroperitoneal adipose tissue, by measuring cell area, perimeter and cellular density. Additionally, the treatments, ameliorated adipose tissue accumulation, insulin resistance, blood pressure, cholesterol and triglycerides concentration in serum, and reduced lipogenesis and inflammation by downregulating acetyl-CoA carboxylase (ACC) gene expression, increasing serum concentration and expression of adiponectin as well as downregulating IL-6 expression. Both fucoxanthin and *Undaria pinnatifida* may be considered for treating obesity and other diseases related.

## 1. Introduction

Nowadays, obesity is one of the most important health issues in the world. According to the WHO, in 2014 more than 1.9 billion adults were overweight, of which 600 million were obese [1]. This is the result of an imbalance between the amount of energy ingested and the energy used [2]. This problem not only results in an excessive accumulation of white adipose tissue (WAT), which could be considered by many as an aesthetic problem, but it also increases health risks by promoting conditions such as diabetes mellitus, heart disease, hypertension, osteoarthritis, gall bladder disease and several types of cancer [3].

Many food ingredients from vegetal and marine sources have been studied to understand their potential and possible beneficial outcomes to health, demonstrating the presence of bioactive compounds with anti-obesity effects. Among them, brown algae (*Phaeophyceae*) have been widely studied because of their composition rich in chlorophylls, sterols, carotenoids, phlorotannins, polyunsaturated fatty acids, polysaccharides (carrageenan, laminarans, fucoidans) showing positive health effects in animal models [4], including activity against hyperglycemia, to improve glucose tolerance, and to have antioxidant, antihypertensive and anti-obesity properties [5,6,7,8]. Several brown algae are currently available for human consumption and are frequently ingested in countries such as Korea or Japan, and among them wakame (*Undaria pinnatifida*) is one of the most important.

One of the compounds from brown algae that has caused great interest because of its anti-obesity properties is the carotenoid fucoxanthin, one of the most abundant carotenoids and found exclusively in brown algae and diatoms (*Bacillariophyta*) [9]. Several in vivo studies have demonstrated that fucoxanthin has a preventive effect on the accumulation of adipose tissue, improving insulin sensitivity, downregulating the secretion of diverse cytokines related to obesity and up- or downregulating genes related to lipid metabolism or energy expenditure. Recent studies showed that supplementation of diet with fucoxanthin downregulates the expression of pro-inflammatory cytokines such as IL-6 and PAI-1 [10] and upregulates the expression of the anti-inflammatory cytokine adiponectin [11]. Fucoxanthin has also beneficial effects on lipid and glucose plasma levels [12] thus reducing hyperinsulinemia [13]. Furthermore, fucoxanthin has been shown to inhibit lipogenesis by downregulating ACC [14] and to promote the browning of WAT by increasing the expression of UCP-1 in this tissue [15].

The aim of the present study was to examine the effect of brown algae *Undaria pinnatifida* and one of its bioactive compounds fucoxanthin on lipid metabolism, inflammation and biomarkers of cardiovascular function in a murine model fed with a high-fat diet. It was concluded that both the algae and the carotenoid have a protective effect that ameliorates several biochemical, physiological and inflammation parameters as well as affecting on the up- or downregulation of several genes related to lipid metabolism and energy expenditure.

## 2. Results

### 2.1. Effect of Undaria pinnatifida and Fucoxanthin on Body Weight Gain, Energy Intake, and Body Fat Mass

**Figure 1:**
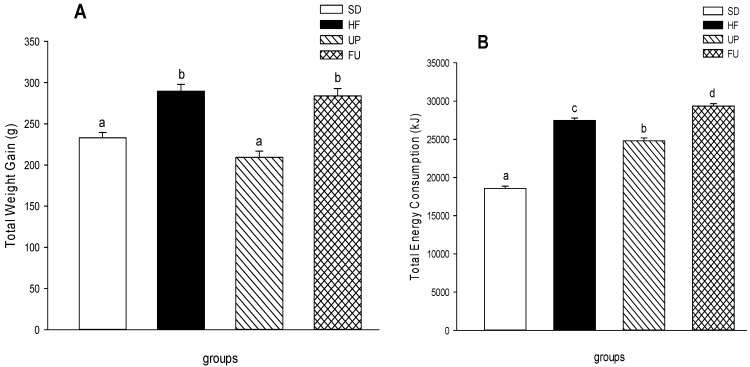

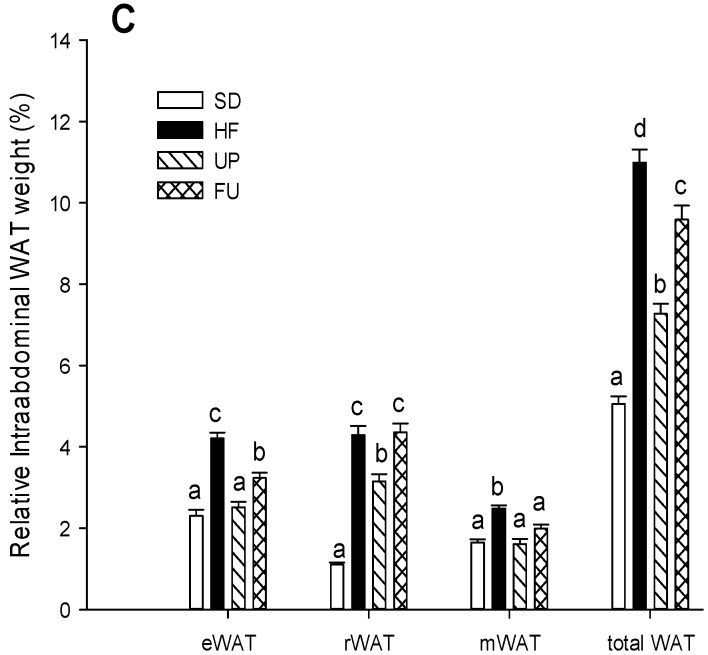
Effect of brown algae *Undaria pinnatifida* and fucoxanthin on (**A**) total body weight gain (g) (**B**) total energy intake (kJ) and (**C**) intraabdominal fat accumulation of white adipose tissue (WAT): epididyml (eWAT), retroperitoneal (rWAT) and mesenteric (mWAT) (%) over a time period of 8 weeks. The groups are abbreviated as: standard diet (SD); high-fat diet (HF); HF + *Undaria pinnatifida* (UP); HF + Fucoxanthin (FU). Values are shown as mean ± SE, (*n* = 6 per group). Data was compared by one-way ANOVA with Holm-Sidak test for multiple comparisons. Means with different superscripts are significantly different (*p <* 0.05).

The total weight gain of the different groups, the final body weight of the animals minus the initial body weight, is shown in Figure 1A. The HF group had a weight gain 1.3 times greater than the SD group, showing a significant difference after the 8 weeks of treatment (*p <* 0.05) due to the diets ingested (standard diet vs. high fat diet). The UP group presented the smallest weight gain with no difference from the SD group despite the high-fat diet consumed, while FU group showed no weight gain reduction compared to the group fed with the high-fat diet (HF).

With respect to food intake, Table 1 shows this information by group through the 8 weeks of the study. We can observe that both control groups, SD and HF, showed no significant difference in food intake (g) despite being fed with different diets. The UP group presented the smallest food intake in some of the weeks of the study, but in some others (and in average) it presented no significant difference with respect to the control groups (SD and HF). Finally FU group showed the greatest food intake only on week two with respect to the other groups; in the rest of the weeks and on average it showed no significant difference with respect to the SD and HF groups, but it showed a greater food intake with respect to the UP group (*p <* 0.05).

Regarding energy consumption, the SD group was fed with a standard diet with caloric density of 3.1 kcal/g while the HF, UP and FU groups were fed with a high-fat diet containing 4.5 kcal/g. Figure 1B shows that the SD group was the one that had less energy intake due to the caloric density of the standard diet supplied to the animals, while the HF group had a greater energy intake because of the caloric density of the high-fat diet. The UP group, despite being fed with the same high-fat diet, showed 8% less energy intake than the HF group (*p <* 0.05), whereas the FU group showed a significant increase in energy intake with respect to the HF group.

In Figure 1C, the percentage of intraabdominal body fat mass with respect to the total final body weight is presented, considering the weight of epididymal, retroperitoneal and mesenteric adipose depots. The SD group had a total intraabdominal fat mass percentage of 5.10%, and as expected the group with the greatest intraabdominal fat mass was the HF group with 11.33% due to the high-fat diet ingested by this study group (*p <* 0.05). UP group showed the lowest percentage of intraabdominal fat mass among the high fat diet groups (7.11%), no difference compared to the SD group was perceived in the epididymal and mesenteric fat depots even though the group of rats was fed with a higher caloric diet and the retroperitoneal fat depot was smaller than the HF group (*p <* 0.05). The FU group had a reduced percentage of total intraabdominal fat mass of 9.35% compared to the group fed with the hypercaloric diet (HF) (*p <* 0.05), and this was also true for its epididymal and mesenteric fat depots. Regarding retroperitoneal fat, it showed no difference with respect to the HF group.

### 2.2. Effect of Undaria pinnatifida and Fucoxanthin on Serum Lipid Profile

In Table 2, we can observe that the rats fed with the high fat diet had increased levels of TG, total and LDL cholesterol and a decreased level of HDL cholesterol compared to the rats fed with the standard diet (SD group). In general both treatments, UP and FU had an important effect on decreasing these serum levels even though they were fed with the same high-fat diet as the HF group.

For triglycerides, the highest level was found in the HF group, 1.9 times greater than the control group (SD). The FU group showed no difference (*p <* 0.05) in the TG concentration compared to the SD group, despite being fed with the hypercaloric diet, while UP group showed an even significantly lower TG level compared with this same group (SD) (*p <* 0.05).

With respect to cholesterol, the HF group had a 1.6-fold higher concentration level than the SD group (*p <* 0.05). The UP group showed no difference on the total cholesterol concentration compared to the SD group. On the other hand, the FU group showed a decrease of 30% compared to the cholesterol serum level of the HF group (*p <* 0.05). Regarding HDL cholesterol serum level, HF group showed the lowest, 23% less than the level obtained for the SD group. SD and UP group showed no difference, and FU group had the most important effect on this parameter with the highest HDL serum level, 40% greater than HF group (*p <* 0.05). In the case of the LDL/VLDL cholesterol level, HF group presented a value 6-fold greater than SD group showing that its main composition of cholesterol is from LDL and VLDL particles. Both UP and FU groups showed significantly lower LDL concentrations than the HF group (*p <* 0.05) with concentrations 4- and 3.4-fold lower respectively.

### 2.3. Effect of Undaria pinnatifida and Fucoxanthin on Glucose Homeostasis and Insulin Resistance

As shown in Table 2, HF group showed the greatest blood glucose and insulin serum levels due to the high fat diet consumed for 8 weeks. Both treatments, UP and FU, attenuated the increases produced by the HF.

For glucose, HF group had a serum concentration 1.6 times greater than SD group. UP group blood glucose concentration showed a 38% reduction (*p <* 0.05) with respect to the high fat diet group (HF), while FU group had a 29% glucose reduction compared to this group (*p <* 0.05) (HF).

Insulin serum level for HF group was 1.5-fold greater than SD group showing again the effect of the HF diet. UP group had the lowest insulin level of all groups (*p <* 0.05) despite the fact of being fed with the high-fat diet, it was even lower than SD group (1.2-fold). FU group showed a lower than HF group insulin level with a reduction of 30%. The HOMA index for HF group was 2.5 times greater than SD group (*p <* 0.05) while UP and FU groups showed a 1.8 and 1.3-fold decrease (*p <* 0.05) compared to HF group.

**Figure 2:**
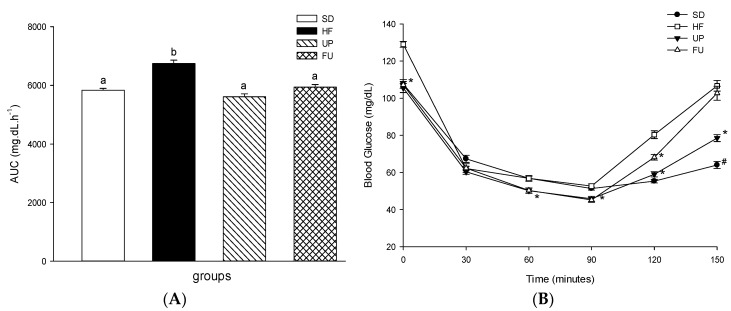
Effect of brown algae *Undaria pinnatifida* and fucoxanthin on insulin resistance. Area under the curve (AUC) (**A**) and insulin tolerance test blood glucose levels during insulin tolerance test (**B**). The groups are abbreviated as: standard diet (SD); high-fat diet (HF); HF + *Undaria pinnatifida* (UP); HF + Fucoxanthin (FU). Values are shown as mean ± SE, (*n* = 6 per group). Data was compared by one-way ANOVA with Holm-Sidak test for multiple comparisons. Means with different superscripts are significantly different (*p* < 0.05). * *p* < 0.05 vs. HF group; # *p* < 0.05 vs. all high fat fed groups (HF, UP, FU).

In order to evaluate the tissues response to insulin, blood glucose levels were also evaluated after injecting insulin to the different groups through 150 min. As shown in Figure 2B, SD group as well as UP and FU had a similar insulin response in the first 90 min of the experiment, while HF group showed a behavior that characterizes insulin resistance. However, at 120 min, FU rats recovered blood glucose to levels similar to those of HF rats. The area under the curve was also calculated (Figure 2A) being greater for the HF group as compared to SD group (*p <* 0.05). In the case of UP and FU groups, they both presented no difference in the area under the curve compared to the SD group despite the high fat diet provided.

### 2.4. Effect of Undaria pinnatifida and Fucoxanthin on Blood Pressure

**Figure 3:**
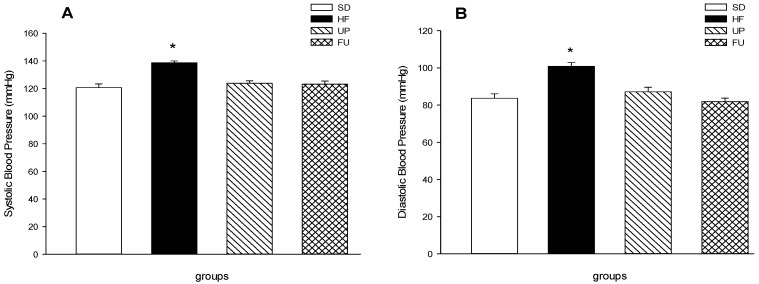
Effect of brown algae *Undaria pinnatifida* and fucoxanthin on blood pressure. Systolic (**A**) and (**B**) diastolic pressure (mmHg) over a time period of 8 weeks. The groups are abbreviated as: standard diet (SD); high-fat diet (HF); HF + *Undaria pinnatifida* (UP); HF + Fucoxanthin (FU). Values are shown as mean ± SE, (*n* = 6 per group). Data was compared by one-way ANOVA with Holm-Sidak test for multiple comparisons. Means with * are significantly different (*p* < 0.05).

As shown in Figure 3A,B, blood pressure in SD groups was within normal values, with a systolic pressure of 120 mmHg and a diastolic pressure of 71 mmHg, while on the other hand, HF group showed higher blood pressure with 139 mmHg of systolic pressure and 101 mmHg of diastolic pressure showing an increase of 15% and 40% respectively than the SD group (*p <* 0.05). Both UP and FU groups showed no difference (*p <* 0.05) neither in systolic nor diastolic pressure compared with SD group, hence having a lower pressure than HF group despite the fact of being fed with the same high-fat diet.

### 2.5. Effect of Undaria pinnatifida and Fucoxanthin on Cytokines in Blood Serum

**Figure 4:**
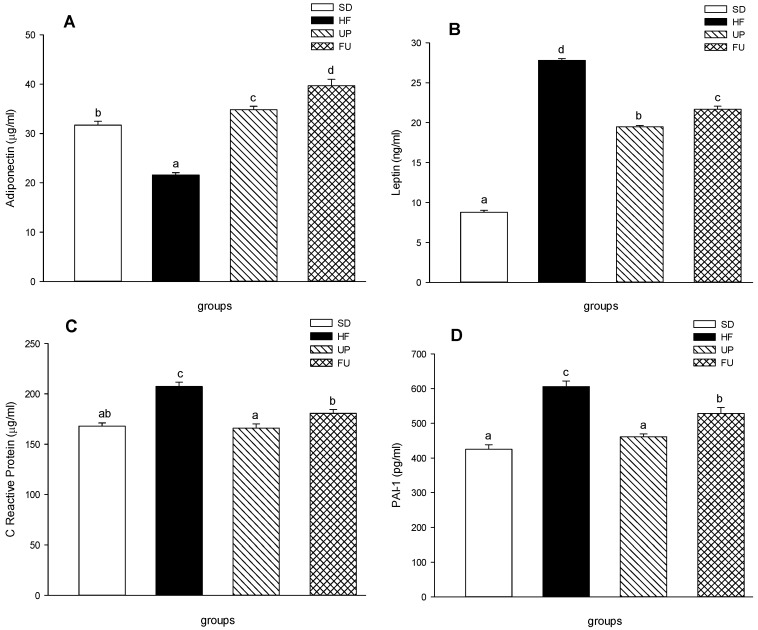
Effect of brown algae *Undaria pinnatifida* and fucoxanthin on (**A**) adiponectin (μg/mL); (**B**) leptin (ng/mL); (**C**) C reactive protein (μg/mL); (**D**) PAI-1 (pg/mL) measured on blood serum. The groups are abbreviated as: standard diet (SD); high-fat diet (HF); HF + *Undaria pinnatifida* (UP); HF + Fucoxanthin (FU). Values are shown as mean ± SE, (*n* = 6 per group). Data was compared by one-way ANOVA with Holm-Sidak test for multiple comparisons. Means with different superscripts are significantly different (*p <* 0.05).

Figure 4 shows the effect of the treatments on anti-inflammatory and pro-inflammatory markers. Both treatments, UP and FU showed a positive effect increasing the anti-inflammatory cytokine adiponectin and decreasing the pro inflammatory cytokines leptin, C reactive protein and PAI-1.

The adiponectin serum levels were 1.5-fold lower in the HF group compared to SD group (*p <* 0.05) (Figure 4A). Although UP and FU groups also were fed with high-fat diet, the treated rats showed an increase in the adiponectin serum levels, 1.6 and 1.9 higher as compared to HF group respectively (*p <* 0.05), being FU the most effective treatment. Serum level of leptin (Figure 4B) in HF group was 3-fold higher than the leptin level found on SD group. UP group had a decrease on leptin level (25%) compared to the HF group, while FU group also showed a 22% reduction on this hormone serum level compared to the HF group, (*p <* 0.05). In Figure 4C, C reactive protein serum level in HF group showed a 20% increase compared to the SD group, due to the high fat diet consumed over the 8 weeks. Both UP and FU groups showed significantly lower serum levels compared to the HF group and no difference with respect to SD group (*p <* 0.05), which also could indicate diminished inflammation in treated rats. PAI-1 serum level (Figure 4D) in HF group was 1.4-fold higher than SD group. UP group PAI-1 concentration was 24% lower than the concentration of the HF group (*p <* 0.05) despite the fact that UP group was fed with a high fat diet. On the other hand FU group PAI-1 serum level was 15% lower than HF group (*p <* 0.05).

### 2.6. Effect of Undaria pinnatifida *and* Fucoxanthin on Gene Expression of Inflammatory Markers

**Figure 5:**
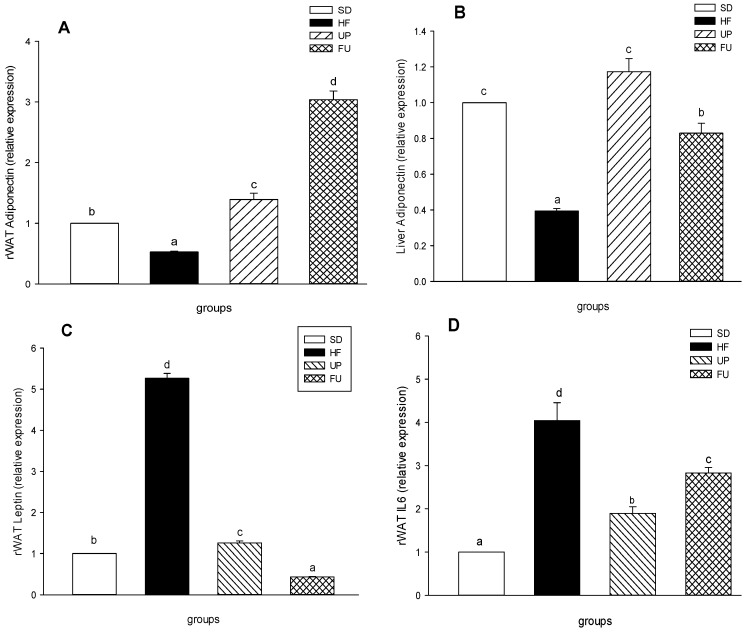
Effect of brown algae *Undaria pinnatifida* and fucoxanthin on cytokine mRNA expression in retroperitoneal adipose tissue (rWAT) and liver. Quantitative real-time PCR assays of the genes encoding (**A**,**B**) adiponectin (**C**) leptin and (**D**) IL-6. Values are expressed as the-fold change compared with SD group that was arbitrarily set to 1. The groups are abbreviated as: standard diet (SD); high-fat diet (HF); HF + *Undaria pinnatifida* (UP); HF + Fucoxanthin (FU). Values are shown as mean ± SE, (*n* = 6 per group). Data was compared by one-way ANOVA with Holm-Sidak test for multiple comparisons. Means with different superscripts are significantly different (*p* < 0.05).

Regarding gene expression, and as seen on Figure 5A, adiponectin expression in retroperitoneal fat (rWAT) showed a 50% reduction on the HF group compared to SD group (*p <* 0.05). Both treatments, UP and FU upregulated the adiponectin gene expression, being FU the one with the best effect by increasing the expression 3 times, followed by UP group with a 1.4-fold greater than the SD group (*p <* 0.05). In liver tissue (Figure 5B), the behavior of this gene was similar regarding HF group having a lower expression than SD group (2.5 times greater). UP and FU groups had 3 and 2 times greater expression levels compared to HF group (*p <* 0.05).

In the case of leptin expression, the level for HF group (Figure 5C) was 5 times greater than SD group. UP and FU groups showed a downregulation of leptin gene expression, with an important decrease compared to HF group although they were also fed with the same high fat diet, the values obtained were 5 and 5.6 times lower (*p <* 0.05). Finally, in Figure 5D, we can see that rWAT IL-6 expression on the HF group was 4 times higher than HF group. UP and FU groups showed a lower expression of this gene compared to HF group (*p <* 0.05) with expression levels 2.1 and 1.4 lower, respectively.

### 2.7. Effects of Undaria pinnatifida and Fucoxanthin on PPARα, PGC1α, and UCP1 Gene Expression

**Figure 6:**
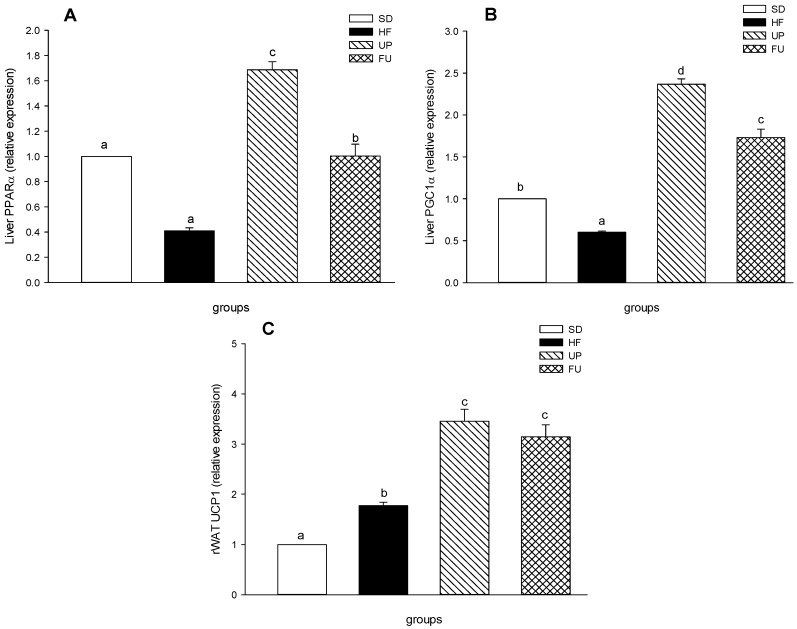
Effect of brown algae *Undaria pinnatifida* and fucoxanthin on expression of genes involving fatty acid oxidation and energy expenditure in retroperitoneal adipose tissue (rWAT) and liver. (**A**) PPARα; (**B**) PGC1α and (**C**) UCP1 were measured by quantitative real time PCR assay. Values are expressed as the-fold change compared with SD group that was arbitrarily set to 1. The groups are abbreviated as: standard diet (SD); high-fat diet (HF); HF + *Undaria pinnatifida* (UP); HF + Fucoxanthin (FU). Values are shown as mean ± SE, (*n* = 6 per group). Data was compared by one-way ANOVA with Holm-Sidak test for multiple comparisons. Means with different superscripts are significantly different (*p* < 0.05).

PPARα and PGC1α expression levels in liver (Figure 6A,B) showed a similar behavior, both of these genes showed a lower expression in HF group than SD group. For genes associated with energy expenditure and fatty acid oxidation (PPARα, PGC1α and UCP1), treatments with UP and FU upregulated the gene expression. UP group showed the greatest PPARα gene expression (70% greater with respect to SD group) and FU group presented no difference with respect to SD group (*p <* 0.05), while for PGC1α, UP, and FU group increased in 2.4 and 1.7-fold the expression of this gene with respect to the SD group (*p <* 0.05). Figure 6C shows a 70% increase in the UCP1 gene expression in HF group vs. SD group. Both UP and FU groups showed no difference between them and presented the highest expression levels for UCP1 (*p <* 0.05) with 3.4 and 3.1 times increases compared to SD group respectively.

### 2.8. Effect of Undaria pinnatifida and Fucoxanthin on PPARγ and ACC Gene Expression

**Figure 7:**
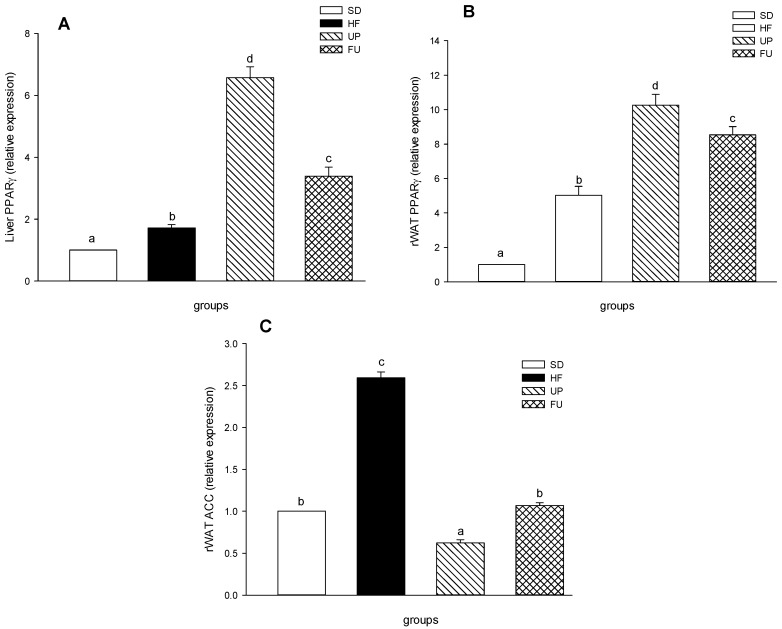
Effect of brown algae *Undaria pinnatifida* and fucoxanthin on expression of genes involved in lipid metabolism in retroperitoneal adipose tissue (rWAT) and Liver (**A**,**B**) PPARγ and (**C**) ACC were measured by quantitative real-time PCR assay. Values are expressed as the-fold change compared with SD group that was arbitrarily set to 1. The groups are abbreviated as: standard diet (SD); high-fat diet (HF); HF + *Undaria pinnatifida* (UP); HF + Fucoxanthin (FU). Values are shown as mean ± SE, (*n* = 6 per group). Data was compared by one-way ANOVA with Holm-Sidak test for multiple comparisons. Means with different superscripts are significantly different (*p* < 0.05).

PPARγ expression in liver and retroperitoneal fat are shown in Figure 7A,B respectively, showing similar changes for both tissues, but with higher expression in fat adipose tissue than liver. In liver (Figure 7A), HF group had 1.7-fold more than SD group, while UP group had the highest expression of all the groups (*p <* 0.05), showing an increase in 6.5 times, followed by FU with 3.4-fold greater than SD group. For adipose tissue (Figure 7B), HF group expression was 4.7 times higher than SD group while UP presented an increase in 10.3-fold. FU also had a higher expression level being 8.5 times greater than SD group.

Figure 7C shows the ACC expression in rWAT, where the HF group was 2.6 times greater than SD group. FU group expression showed no difference with respect to SD group (*p <* 0.05) while UP group expression was the lowest (*p <* 0.05), being 60% compared to SD group.

### 2.9. Effect of Undaria pinnatifida and Fucoxanthin on Adipocyte Size and Cellular Density

**Figure 8:**
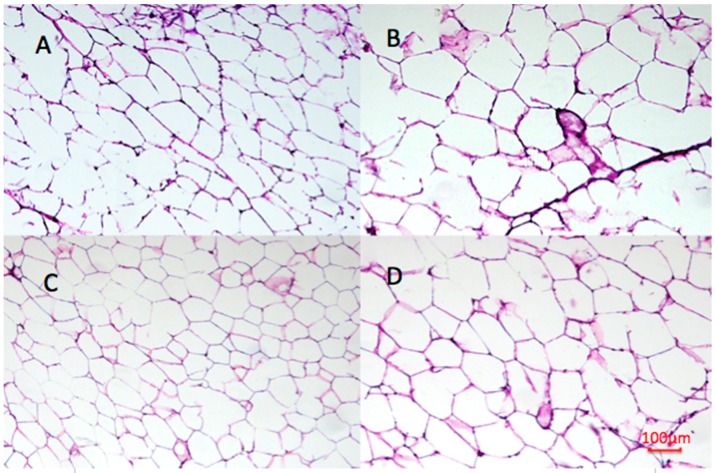
Effect of *Undaria pinnatifida* and fucoxanthin on adipocytes from retroperitoneal adipose tissue. Histological features of (**A**) SD group; (**B**) HF group; (**C**) UP group and (**D**) FU groups are presented.

**Figure 9:**
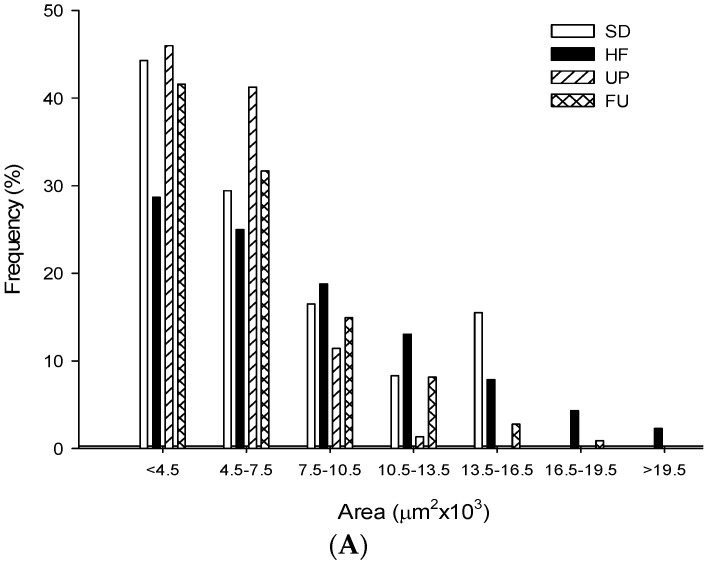

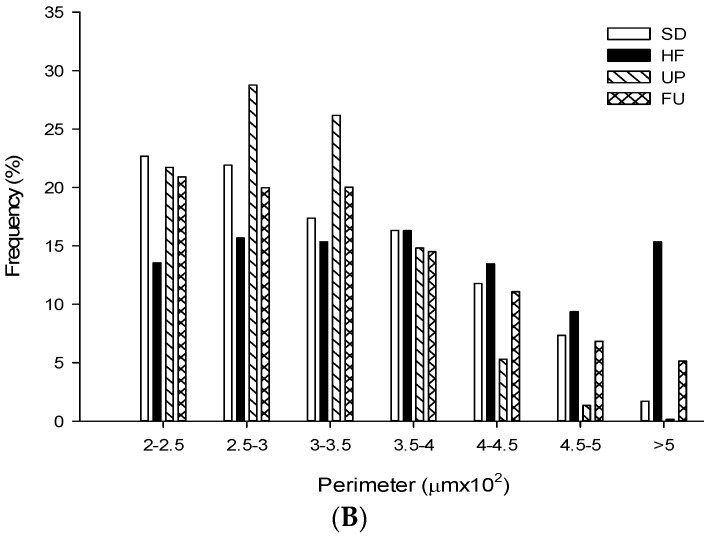
Frequency distribution of adipocyte (**A**) area and (**B**) perimeter from SD, HF, UP, and FU groups.

With respect to the image analysis performed on retroperitoneal adipose tissue, and as shown in Table 3, we could observe in Figure 8 that the HF group showed bigger adipocytes (40% bigger) than the SD group with respect to the area obtained. The FU group showed no difference for this parameter compared to the SD group (*p <* 0.05) and UP had the smallest adipocytes of all the groups being 15% and 42% smaller than the SD and HF groups respectively. These differences are observed in the micrographics obtained shown in Figure 8. Also regarding the cell size, the perimeter was evaluated. HF group had a 1.15-fold in perimeter than SD group; UP group as well as having the smallest area had the smallest perimeter being 24% smaller than HF group while FU group also had a smaller perimeter than HF group, 11% smaller (*p <* 0.05). In Figure 9, the frequency of size distribution is shown in order to represent the different sizes of adipocytes obtained in the study. Regarding cellular density, SD group showed 1.46 times more cells per mm^2^ compared to HF group. FU group had no difference regarding cellular density compared to SD group, and UP group had the greater number of cells per mm^2^, with 78% and 21% more than HF and SD groups, respectively.

## 3. Discussion

In this study we provide evidence that both *Undaria pinnatifida* and fucoxanthin have beneficial effects on obesity and associated metabolic disorders in rats fed with a hyperlipidic diet. The results from the current study showed that the UP group exhibited a protective effect on body weight gain despite being fed with a high fat diet (21% fat); this result was similar to the results reported by Tong et al. in which the same dose of seaweed was used [7], and no significant difference was observed on body weight of FU group. The effect of FU on body weight is still controversial. Several authors have reported that FU had a beneficial effect on body weight gain in mice or rats when fed with a high-fat diet [16,17] but others have stated that no significant difference was shown [6,18,19,20], the doses used for the purpose of these studies were much bigger than the one used for this experiment and the duration of treatment was also different. In this study, the fucoxanthin present in *Undaria pinnatifida* does not directly cause reduction in body weight, suggesting that the other components of algae, such as eicosapentaenoic acid (EPA), and fiber are responsible for that effect. The effect of PUFAS on reducing body weight, especially the combination of Ω3 and Ω6, is well known [21]. Regarding WAT accumulation, fucoxanthin showed a positive effect (*p <* 0.05). Both groups, UP and FU had significantly lower mesenteric, retroperitoneal and epididymal fat pads, and it is important to note that the mesenteric fat pads showed no difference (*p <* 0.05) compared to the group fed with the standard diet. Several studies have shown the attenuating effect of fucoxanthin and algae on WAT mass, some of them even focusing on retroperitoneal, epididymal and visceral fat [6,7,17,18,19]. Fucoxanthin in brown algae is showing a protective effect on WAT accumulation, and in the case of the algae the other compounds together with fucoxanthin are showing a synergic effect making fat accumulation even less.

TG and cholesterol serum levels had a significant decrease observed in the UP and FU groups regarding total cholesterol, LDL-VLDL cholesterol and TG, and a significant increase was observed regarding HDL cholesterol compared to HF group. In previous studies in humans which had a diet supplemented with different types of algae, a decrease was observed in TG and HDL cholesterol in serum, although no significant change was observed regarding total cholesterol and LDL cholesterol; this effect was attributed to the high amount of fiber present in this algae [22], and the same could be said for our results in which the algae showed better results than the fucoxanthin alone. Regarding FU group, it also showed an improvement in serum lipid profile compared to the high-fat fed group. Some studies using different doses of fucoxanthin have also shown a protective effect of this carotenoid against lipid accumulation in serum, resulting especially in lower TG levels and higher HDL cholesterol levels [23,24], produced by the modulation of genes involved in lipid metabolism such as SREBP-1c, CPT1, CYP7A1 which were not explored in this paper. Additionally, the diminished expression of ACC observed in WAT tissue of both treatments, could be an indicator of reduced de novo fatty acid synthesis, as has been previously reported [25].

Insulin sensitivity is diminished when high-fat diets are consumed, which was observed in the HF group. Both treatments showed a protective effect possibly linked with an increase in the concentration of serum adiponectin, together with an upregulation in the expression of the gene related to this adipokyne. This is also closely associated with insulin sensitivity by regulating IRS-1 and IRS-2 on liver [26]. Other studies have been made with brown algae and fucoxanthin in which serum glucose levels and insulin resistance were reduced by the activation of MAPK pathway or simply by avoiding fat absorption because of high fiber content in algae [5,13,27].

Another physiological parameter that is altered by high-fat diet ingestion is blood pressure. In this experiment, it was observed that both treatments had a positive effect on both diastolic and systolic blood pressure. As we could observe for this parameter, both treatments showed no significant difference with each other; the algae contains a lower quantity of fucoxanthin (2.5 times) so other components present on the algae contribute to this effect. Some reports indicate that *Undaria pinnatifida* contains fatty acid EPA [28,29] that has been associated with diminishing blood flow and anti-inflammatory properties [29] associated to this condition. Also, previous studies but using different types of algae, have also proven the effectiveness of algae in reducing this parameter [30] by containing antioxidant molecules, fibers such as fucoidans, and by inhibiting the action of angiotensin converting enzyme [31,32]. Blood pressure is also linked to PAI-1 serum concentration, and this molecule acts as a pro inflammatory cytokine, and has been linked to thrombosis and fibrosis [10] which was diminished by treatments and could also explain the decrease on this parameter. To our knowledge, this is the first article that approaches cardiovascular health markers related to the ingestion of fucoxanthin and *Undaria pinnatifida*, showing a beneficial effect in both treatments.

In obesity, a higher concentration of pro-inflammatory cytokines and a lower of anti-inflammatory is a consequence of the chronic low-grade inflammation state, which characterizes this pathology. When WAT tissue is incremented, the secretion of pro inflammatory molecules is increased, as was seen on the HF group. The treatment groups (UP and FU), showed less WAT tissue and by consequence less concentration and less expression of pro-inflammatory citokynes (IL-6, C-reactive protein, PAI-1). Both PAI-1 and IL-6 have been studied with regard to the treatment of high fat diets using algae or fucoxanthin, and the same effect as our study was obtained [10,33]. Another protein of interest related to inflammation is C-reactive protein. It is a hepatic acute phase protein that is regulated by the levels of IL-6 present in the circulation. It has been linked to infection and in the last years to more complex chronic inflammation diseases such as obesity, insulin resistance or endothelial dysfunction [34]. This protein has not been thoroughly evaluated related to obesity and not many reports are available with respect to the effect of brown algae or fucoxanthin on this parameter. In our study we could observe that C-reactive protein in serum is diminished by the action of both treatments (UP and FU) with respect to the control group fed with the same high-fat diet which could be indicative of other effects observed in this study such as increased insulin sensitivity or ameliorated blood pressure. Another hormone associated with obesity is leptin. This hormone concentration is diminished during fasting and after food consumption is produced by the adipose tissue. Leptin sends a signal to the hypothalamus to inhibit appetite; in obesity, a leptin resistance state has been found and the serum levels are increased [35]. In our study we found that both the serum concentration and the expression were increased in a state of excessive WAT accumulation; on the other hand, with the treatments applied and as several authors have reported, serum level and expression of leptin were decreased [12,16,17,20]. Finally, adiponectin, a cytokine associated with anti-inflammatory processes, has been found to be increased on normal weight individuals and decreased on obesity; it also promotes fatty acid oxidation, increases insulin sensitivity and energy expenditure [36]. Adiponectin serum level concentration and expression was increased by the effect of the treatments administered to the study groups, UP and FU indicating a beneficial effect on the high-fat diet fed animals.

Another mechanism for ameliorating WAT accumulation and improving energy expenditure was presented when UP and FU groups had an important effect on the expression of UCP-1 compared to HF and SD groups. This effect has been previously evaluated in mice that were fed with 0.2% of fucoxanthin [18]. This point is really important together with preadipocyte differentiation because it is an indicative of browning of WAT. The new adipocytes or existing adipocytes could be more similar to brown fat than to white, having the ability of performing β oxidation and to generate heat [37] avoiding body weight gain. Also regarding energy expenditure we find a higher expression of both PPARα and PGC1α in the liver and are responsible for homeostasis regulation as they increased energy expenditure by promoting fatty acid oxidation and mitochondrial biogenesis instead of expanding adipose tissue [38]. Both treatments showed an increase in the expression of these genes, showing a state of greater energy expenditure (compared to HF group), which could explain a lower adipose tissue on the treated groups in which the fat consumed by diet was oxidized instead of stored. Only another previous study was found regarding these genes in which the expression was measured in retroperitoneal WAT and brown adipose tissue, after supplementing mice diet with fucoxanthin, resulting after 5 weeks a significant increase in the expression of PGC1α in the retroperitoneal WAT and also an increase in the expression of PPARα of brown adipose tissue [19]. To our knowledge no other studies have been made with respect to these genes expression in liver and the treatment with fucoxanthin nor *Undaria pinnatifida*.

Regarding the expansion of adipose tissue, peroxisome-proliferator-activated receptor γ (PPARγ) has an important role controlling adipose tissue expansion when positive energy balance is presented (such as in high-fat diet ingestion). This topic has had its controversies, since some authors declare that this receptor is downregulated by the action of fucoxanthin and that the pre-adipocyte differentiation is inhibited [39] while other studies concluded that PPARγ is upregulated by the action of fucoxanthin [19]. In our study, we observed an upregulation of this receptor and preadipocyte cell differentiation suggested by the different size of the adipocytes obtained from microscopy analysis. As it was shown adipose cells on UP and FU groups showed a smaller diameter and area, together with a greater cell number per area compared to the HF group. PPARγ has been proved to promote apoptosis of large hypertrophic mature adipocytes and to stimulate instead the production of small insulin sensitive adipocyte, also playing an important role in insulin sensitivity [40,41].

The expression of PPARγ in liver in response to the administration of algae or fucoxanthin has not been thoroughly studied. Several studies have demonstrated that a high-fat diet has an impact on upregulating this gene, and this has been associated with fat accumulation in the liver [42]. Regarding glucose homeostasis, when PPARγ is normally expressed on adipose tissue the impact of this gene on the liver is minimal or nonexistent [41]. In our study we found that both treatments resulted in a greater expression of this gene even compared to the HF group. This result could be controversial since a previous study concluded that fucoxanthin supplementation in mice downregulated the expression of this gene in liver [12]. Nevertheless, several studies have been performed to understand the role of this receptor in liver tissue. Gavriola et al. [43] concluded that mice that were knocked out for PPARγ, specifically in the liver, had worsened hyperlipidemia, triglyceride clearance and muscle insulin resistance when compared to mice with the gene normally expressed. This could explain why our study groups showed beneficial effects in terms of glucose plasma levels, insulin resistance and diminished hyperlipidemia when compared to HF group. Also an increase in PPARγ on liver has been proved to stimulate fat accumulation in other areas such as subcutaneous rather than visceral depots and to diminish circulating lipids [44], which could also explain the improvements on these parameters presented in this paper. The effects of UP and FU treatments should be studied in more detail in order to understand the mechanisms involved for the increased expression of this receptor specifically in liver tissue.

## 4. Materials and Methods

### 4.1. Materials

Fucoxanthin (F6932; ≥95%) was purchased from Sigma-Aldrich Co. Ltd. (Toluca, Mexico). *Undaria pinnatifida* (cut wakame dried seaweed, Tetsujin) was purchased from Kume Importaciones S.A de C.V. (Ciudad de México, Mexico).

### 4.2. Rat Model

All animal experiments were conducted in accordance with the Escuela Nacional de Ciencias Biológicas Ethics Code for Animal Studies (ENCB-IPN, Mexico) and the Mexican Council for Animal Care Guide for Care and Use of Laboratory Animals (NOM-062-ZOO-1999). Six-week-old male Wistar rats (180 ± 5 g of body weight) were purchased from the Animal House of Autonomous Metropolitan University, Xochimilco Campus (Ciudad de México, Mexico) and individually housed in stainless steel cages with mesh bottoms at 24 ± 2 °C and 40%–60% humidity, on a 12-h light (8:00 A.M.–8:00 P.M.) dark (8:00 P.M.–8:00 A.M.) cycle. The rats were maintained with free access to water and food (standard diet commercially available: Teklad Global 18% Protein Rodent Diet 2018) for 1 week in order to acclimatize. Rats were weighed and divided into 4 groups (*n* = 6) as follows: standard diet control group (SD), high fat diet control group (HF), HF + intragastric administration of *Undaria pinnatifida* (UP, 400 mg/kg bw), HF + intragastric administration of Fucoxanthin (Fu, 1 mg/kg bw). The intragastric administration was provided in a water: tween 80 (100:1) solution which assured total solubility of fucoxanthin and homogeneous dispersion of *Undaria pinattifida* (for Fu and UP groups); also SD and HF groups were given intragastric administration of this water: tween 80 solution. The content of fucoxanthin in *Undaria pinnatifida* was 0.98 ± 0.03 mg/g dry weight, determined by HPLC according to Mendiola et al. [45]. Rats had free access to water and diet for 8 weeks and were administered orally, once daily. Standard diet (Teklad Global 18% protein: 2018), with energy density of 3.1 kcal/g, 18.6% of total protein, 6.2% of fat and 44.2% of carbohydrate was purchased from Harlan Laboratories, Inc. (Teklad Global Diets, Madison, WI, USA). High-fat diet with an energy density of 4.5 kcal/g, 17.3% of total protein, 21.2% of fat and 48.5% of carbohydrate was prepared in our laboratory according to Table 4.

Individual body weight and food intake was recorded every day. Energy intake was calculated by multiplying feed mass by its respective caloric density factor. At the end of week 8 rats were fasted for 12 h and euthanized with pentobarbital sodium (32 mg/kg bw). Blood samples (5 mL) were collected from abdominal aorta into sterile gold BD Vacutainer SSTTM test tubes (Cat. No.: 367983, Franklin Lakes, NJ, USA), allowed to clot 15 min and centrifuged at 1465× *g* for 15 min to obtain serum. Retroperitoneal, mesenteric and epididymal fat and liver were removed, rinsed with 1× Phosphate Buffered Saline (PBS Buffer), weighed, frozen in liquid nitrogen and stored at −80 °C until further analyses.

### 4.3. Biochemical Analysis

At week 8, rats were overnight fasted and blood glucose levels were measured in venous blood drawn from the tail vein using a glucometer (ACCU-CHEK^®^ Performa, Roche Diagnostics, Indianapolis, IN, USA). Levels of triacylglycerols and cholesterol were determined in blood serum with enzymatic assays using commercially available kits (cat. 1001310, 1001090; Spinreact, Girona, Spain) and measured in a semi-autoanalyser (Ekem Plus control Lab, Mindray, Shenzhen, China). HDL and LDL-VLDL serum levels were measured by fluorometric method using a commercial available kit (cat. ab65390; Abcam, Cambrige, UK).

### 4.4. Measurement of Insulin Sensitivity and Insulin Serum Levels

Insulin sensitivity was measured according to Barrientos et al. [46] at week 8. Rats were fasted for 5 hours and blood glucose was measured from the tail vein using a glucometer (ACCU-CHEK^®^ Performa, Roche Diagnostics, Indianapolis, IN, USA). After that, 0.6 IU/kg bw of human insulin (Humulin R; Eli Lilly, Ciudad de México, Mexico) was injected and blood glucose was measured at 30, 60, 90, 120, and 150 min. The area under the curve was calculated. Quantitative estimations of insulin serum levels (cat. EZRMI-3K; Millipore, St. Charles, MO, USA) was done following the manufacture’s protocols using a commercial ELISA kit.

### 4.5. Blood Pressure Measurement

At the end of week 7 of treatment, systolic and diastolic blood pressure were measured on the conscious restrained rat warmed to 36 °C in a noise free environment by the tail-cuff method using semi-automated equipment (IITC; Life Science Instruments, Woodland Hills, CA, USA). The animals underwent trough an adaptation process of 7 days before the measures were performed.

### 4.6. Cytokine Levels in Blood Serum

Quantitative estimations of leptin (cat. EZRL-83K; Millipore, St. Charles, MO, USA), adiponectin (cat. EZRADP-62K, Millipore, MO, USA), C-reactive protein (cat. CYT294; Millipore, St. Charles, MO, USA) and PAI-1 serum levels (cat. Ab198509, Abcam, Cambrige, UK) were performed following the manufacture’s protocols using specific ELISA kits.

### 4.7. RNA Extraction

Total RNA was extracted from retroperitoneal adipose tissue and from liver using TRIzol^®^ reagent according to the protocol provided by the manufacturer (Life Technologies, Carlsbad, CA, USA). Total RNA quantity (OD-260) and quality (OD-260/OD-280) were determined using GENESYS 10 Series (ThemoSpectronic, Thermo Fisher Scientific, Inc., Waltham, MA, USA). The integrity of the RNA was evaluated using denaturing agarose gel electrophoresis. 5 μg of RNA were separated on a 1% agarose gel with ethidium bromide in MOPS buffer, these two contained 0.2 M formaldehyde.

### 4.8. Real Time PCR

Reverse transcription of 1 μg total RNA was performed using Transcriptor First Strand cDNA Synthesis kit (Roche Diagnostics) in the presence of random hexamers. RT reactions were carried out in a thermal cycler (Eppendorf Mastercycler, Hamburg, Germany). The amplified cDNA was quantified using the spetrophotometric method at 260 nm. Real-time reaction mixture (20 μL) contained 1X Light Cycler TaqMan Master (Roche Diagnostics, Mannheim, Germany), 200 nM forward primer, 200 nM reverse primer, 100 nM hydrolysis probe (Rat Universal Probe Library, Roche Diagnostics, Mannheim, Germany), 0.5 U LightCycler Uracil-DNA glyucosylase, and 2 μL cDNA. The ACC primers were 5′ GATCCC CATGGCAATCTG 3′ as the forward primer and 5′ ACAGAGATGGTGGCTGATGTC 3′ as the reverse primer. The IL-6 primers were 5′ ACAACATCAGTCCCAAGAAGG 3′ as the forward primer and 5′ CCTTCAGGAACAGCTATGAA 3′ as the reverse primer. Leptin was detected using 5′ AATGAAGTCCAAACCGGTGA 3′ as the forward primer and 5′ CCAGGATCAATGACATTTCACA 3′ as the reverse primer. For PGC-1α 5′ GGGTCATTTGGTGACTCTGG 3′ and 5′ GCAGTCGCAACATGCTCA 3′ were used as forward and reverse primers respectively. PPARα was detected using 5′ TTTAGAAGGCCAGGACGATCT 3′ as forward primer and 5′ GCACTGGAACTGGATGACAG 3´as reverse primer, and the primers used for PPARγ detection were 5′ GGGGGTGATATGTTTGAACTTG 3′ as forward primer and 5′ CAGGAAAGACAACAGACAAATCA 3′ as reverse primer. For UCP1, the forward and reverse primers were 5′ TGGCCTTCACCTTGGATCT 3′ and 5′ GCCTGCCTAGCAGACATCAT 3′ respectively. The PCR amplification was performed in borosilicate glass capillaries using the Light Cycler Nano Real-Time PCR System (Roche Diagnostics, Mannheim, Germany). The conditions for the assay were an initial incubation at 95 °C for 10 min, followed by 40 cycles of denaturation at 95 °C for 10 s, annealing at 50 °C for 30 s, and extension at 72 °C for 1 min. The relative expression levels of the target genes mRNA were normalized to 18S mRNA levels. The-fold change or relative quantification in gene expression was determined using the 2−ΔΔCT method [47]. The results were expressed in relation to the average expression of the SD group.

### 4.9. Histological Analysis of WAT

Retroperitoneal adipose tissue was removed from animals, frozen in liquid nitrogen and stored at −85 °C until used. Adipose tissue was embedded in Tissue Freezing Medium (cat. 14020108926, Leica Instruments, Nussloch, Germany) and then fifteen micrometer tissue sections were cut in a Freezing Microtome (CM1850, Leica, Nussloch, Germany), placed on slides, stained with crystal violet 1% and mounted with Entellan^®^ (cat. 107961, Merck Millipore, St. Charles, MO, USA). The images were obtained using an inverted optical microscope (Ti-U, Nikon Eclipse, Tokyo, Japan) with a X10 objective.

### 4.10. Image Analysis

The images obtained were analyzed using Image J (Version 2.0.0-rc-44/1.50e) through a series of steps previously reported by Galarraga [48] with minor modifications. The color image was transformed to 8-bit format and then a morphological filter was applied. Then, the image was binarized using Otsu’s automatic thresholding method followed by a binary process of filling holes and watershed. Afterwards a seeded Watershed algorithm was applied to the image and colors were inverted. The objects that touched the image borders were deleted and the remaining objects were measured (number, diameter, area, and Ferret diameter). Finally the objects left in the image were labeled according to their sizes and all the information obtained was later analyzed.

### 4.11. Statistical Analysis

The data presented, are expressed as mean values ± SE, in the case of image analysis, data are presented as mean values ± SD. Statistically significant differences between the groups were determined by one way ANOVA, followed by the Holm-Sidak test for multiple comparisons. A value of *p <* 0.05 represents a significant difference. The software used was Sigma Plot V. 12.0.

## 5. Conclusions

In conclusion, the results provide evidence that both *Undaria pinnatifida* and Fucoxanthin have beneficial effects on obesity and associated metabolic disorders in rats fed with a hyperlipidic diet. Fucoxanthin proved to decrease WAT mass, decrease serum TG, considerably increase HDL cholesterol in serum, improve insulin resistance, diminish blood pressure, increase expression and serum levels of adiponectin, decrease the expression of leptin and promote β oxidation by increasing the expression of UCP-1. On the other hand, *Undaria pinnatifida* had a positive effect on body weight gain, energy consumption, glucose and insulin serum levels, and serum levels of PAI-1; decreased expression of IL-6; increased energy expenditure by upregulating the expression of PPARα, PGC1α, decreased lipogenesis by downregulating ACC; and upregulating PPARγ which resulted in more and smaller adipocytes in retroperitoneal tissue. The study in general proved that these treatments have the ability to improve WAT function in states of positive energy balance by activating mechanisms to prevent excessive lipid accumulation and to reduce inflammation and insulin resistance.

## Figures and Tables

**Table 1:** Effect of *Undaria pinnatifida* and fucoxanthin on weekly food intake.

Weekly Food Consumption (g)				
Week	SD	HF	UP	FU
1	198.12 ± 5.92 ^a^	204.17 ± 6.99 ^a^	183.73 ± 4.14 ^a^	231.77 ± 3.48 ^b^
2	184.18 ± 5.84 ^b^	180.30 ± 6.95 ^b^	153.73 ± 3.67 ^a^	207.20 ± 3.75 ^c^
3	188.48 ± 4.33 ^b^	183.76 ± 5.34 ^b^	152.07 ± 3.34 ^a^	190.57 ± 5.57 ^b^
4	182.70 ± 4.69 ^ab^	183.16 ±4.57 ^b^	157.48 ± 3.89 ^a^	170.93 ± 4.05 ^ab^
5	177.45 ± 4.31 ^ab^	174.47 ± 5.08 ^ab^	167.27 ± 3.91 ^a^	189.08 ± 5.73 ^b^
6	176.80 ± 5.47 ^a^	176.48 ± 3.87 ^a^	173.85 ± 4.53 ^a^	195.12 ± 5.17^a^
7	166.88 ± 2.23 ^a^	168.28 ± 4.06 ^ab^	162.62 ± 4.25 ^a^	188.77 ± 5.43 ^b^
8	164.22 ± 5.24 ^ab^	188.15 ± 4.77 ^b^	166.88 ± 3.73 ^a^	185.08 ± 5.32 ^ab^
Average	179.85 ± 3.92 ^ab^	182.35 ± 3.81 ^ab^	164.70 ± 3.76 ^a^	194.81 ± 6.37 ^b^

Note: The data shown represent means ± SE (*n* = 6 per group). SD, standard diet; HF, high-fat diet; UP, HF + *Undaria pinnatifida*; FU, HF + fucoxanthin. Values with different letters in the same line are significantly different (*p <* 0.05)*.*

**Table 2:** Effect of *Undaria pinnatifida* and fucoxanthin on biochemical parameters.

Biochemical Parameters	SD	HF	UP	FU
Triacylglycerides (mg/dL)	80.38 ± 2.05 ^b^	153.76 ± 3.41 ^d^	69.50 ± 1.97 ^a^	89.12 ± 2.09 ^c^
Cholesterol (mg/dL)	54.67 ± 2.04 ^a^	90.74 ± 1.85 ^c^	53.93 ± 0.95 ^a^	61.69 ± 1.29 ^b^
HDL-Cholesterol (mg/dL)	37.58 ± 0.25 ^b^	28.73 ± 0.73 ^a^	36.46 ± 0.39 ^b^	40.51 ± 0.59 ^c^
LDL/VLDL-Cholesterol (mg/dL)	9.35 ± 0.34 ^a^	55.36 ± 0.84 ^d^	13.64 ± 0.46 ^b^	16.20 ± 0.20 ^c^
Glucose (mg/dL)	83.83 ± 2.01 ^a^	139.5 ± 2.08 ^c^	86 ± 3.17 ^a^	99.5 ± 3.15 ^b^
Insulin (ng/mL)	3.49 ± 0.13 ^b^	5.21 ± 0.20 ^d^	2.93 ± 0.07 ^a^	3.99 ± 0.15 ^c^
HOMA	20.79 ± 0.69 ^a^	51.59 ± 1.39 ^c^	18.04±1.09 ^a^	28.23±1.31 ^b^

Note: The data shown represent means ± SE (*n* = 6 per group). SD, standard diet; HF, high-fat diet; UP, HF + *Undaria pinnatifida*; FU, HF + fucoxanthin. Values with different letters in the same line are significantly different (*p <* 0.05)*.*

**Table 3:** Effect of *Undaria pinnatifida* and fucoxanthin on adipocyte size and cellular density.

Groups	Area (μm^2^ ×10^3^)	Perimeter (μm ×10^2^)	Ferret (μm ×10^2^)	Cellular Density (# cells/mm^2^)
SD	5.79 ± 2.96 ^b^	3.25 ± 0.84 ^b^	1.19 ± 0.33 ^b^	163.79 ± 9.89 ^b^
HF	8.02 ± 4.67 ^c^	3.74 ± 1.11 ^d^	1.31 ± 0.38 ^c^	111.78 ± 9.53 ^a^
UP	5.05 ± 2.00 ^a^	3.01 ± 0.61 ^a^	1.08 ± 0.23 ^a^	198.94 ± 17.92 ^c^
FU	6.01 ± 3.37 ^b^	3.33 ± 0.92 ^c^	1.21 ± 0.34 ^b^	160.65 ± 15.09 ^b^

Note: The data shown represent means ± SD. SD, standard diet; HF, high-fat diet; UP, HF + *Undaria pinnatifida*; FU, HF + Fucoxanthin. Values with different letters in the same column are significantly different (*p <* 0.05).

**Table 4:** Composition of high-fat diet.

Component	Composition (g/kg diet)
Sucrose	340.0
Milk fat	210.0
Casein	195.0
Corn Starch	140.5
Cellulose	50.0
Mineral Mix ^1^	43.0
Vitamin Mix ^2^	15.0
DL Methionine	3.0
Choline	2.0
Cholesterol	1.5

Note: ^1^ Mineral Mix (AIN-93 G-MX); ^2^ Vitamin MIx (AIN-93-VX).

## Abbreviations

The following abbreviations are used in this manuscript:

ACC	Acetyl Co-A carboxylase
CPT1	Carnitine palmitoyltransferase 1
CYP7A1	Cholesterol 7 alpha-hydroxylase
EPA	Eicosapentaenoic acid
HDL	High density lipoprotein
IL-6	Interleukin 6
IRS	Insulin receptor substrate
LDL	Low density lipoprotein
MAPK	Mitogen activated protein kinase
PGC1α	PPARγ coactivator 1α
PPARα	Peroxisome-proliferator-activated receptor α
PPARγ	Peroxisome-proliferator-activated receptor γ
SREBP-1c	Sterol regulatory element-binding protein 1c
TG	Triglycerides
UCP-1	Uncoupling protein 1
VLDL	Very low density lipoprotein
WAT	White Adipose Tissue
